# Supplementary material for: SNP- and Haplotype-Based GWAS of Flowering-Related Traits in Brassica napus
Source: Plants (Basel). 2021 Nov 16;10(11):2475. doi: 10.3390/plants10112475 (PMC8619824; doi:10.3390/plants10112475)
Supplement: Supplementary file 1 [file plants-10-02475-s001.zip › plants-1416699-supplementary.pdf]

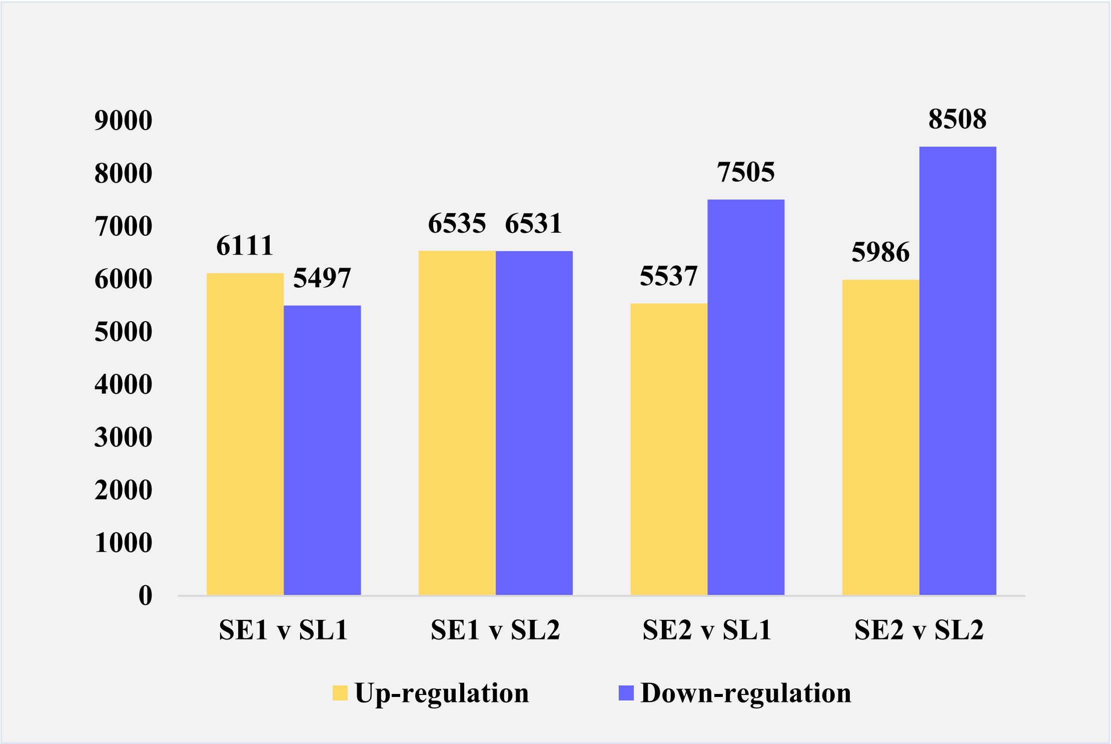

Figure S1. Total number of DEGs (up and down regulation) between early and late flowering samples

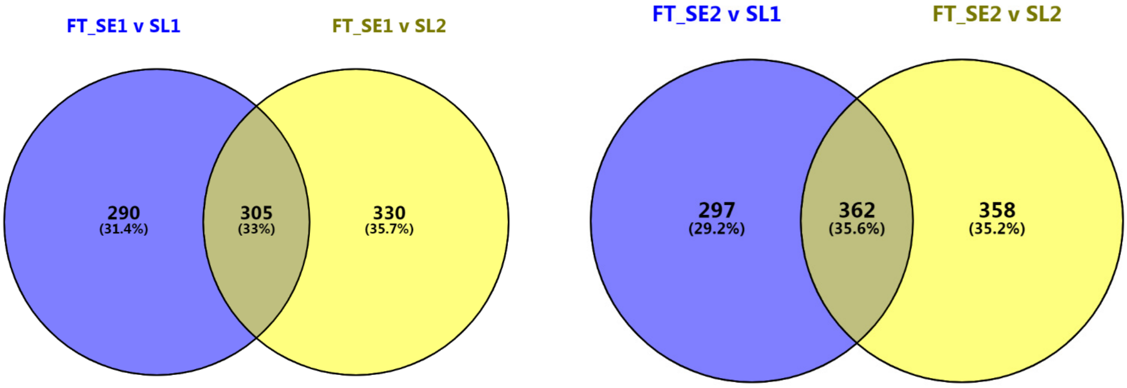

Figure S2. The statistics of DEGs related to flowering time of four samples shown in Venn diagram
